# The Exercising Brain: An Overlooked Factor Limiting the Tolerance to Physical Exertion in Major Cardiorespiratory Diseases?

**DOI:** 10.3389/fnhum.2021.789053

**Published:** 2022-01-21

**Authors:** Mathieu Marillier, Mathieu Gruet, Anne-Catherine Bernard, Samuel Verges, J. Alberto Neder

**Affiliations:** ^1^Laboratory of Clinical Exercise Physiology, Queen’s University and Kingston General Hospital, Kingston, ON, Canada; ^2^HP2 Laboratory, INSERM U1300, Grenoble Alpes University, Grenoble, France; ^3^IAPS Laboratory, University of Toulon, Toulon, France

**Keywords:** exercise tolerance, hypoxia, brain, muscle fatigue, oxygen, respiratory disorders

## Abstract

“Exercise starts and ends in the brain”: this was the title of a review article authored by Dr. Bengt Kayser back in 2003. In this piece of work, the author highlights that pioneer studies have primarily focused on the cardiorespiratory-muscle axis to set the human limits to whole-body exercise tolerance. In some circumstances, however, exercise cessation may not be solely attributable to these players: the central nervous system is thought to hold a relevant role as the ultimate site of exercise termination. In fact, there has been a growing interest relative to the “brain” response to exercise in chronic cardiorespiratory diseases, and its potential implication in limiting the tolerance to physical exertion in patients. To reach these overarching goals, non-invasive techniques, such as near-infrared spectroscopy and transcranial magnetic stimulation, have been successfully applied to get insights into the underlying mechanisms of exercise limitation in clinical populations. This review provides an up-to-date outline of the rationale for the “brain” as the organ limiting the tolerance to physical exertion in patients with cardiorespiratory diseases. We first outline some key methodological aspects of neuromuscular function and cerebral hemodynamics assessment in response to different exercise paradigms. We then review the most prominent studies, which explored the influence of major cardiorespiratory diseases on these outcomes. After a balanced summary of existing evidence, we finalize by detailing the rationale for investigating the “brain” contribution to exercise limitation in hitherto unexplored cardiorespiratory diseases, an endeavor that might lead to innovative lines of applied physiological research.

## Introduction

It has long been argued that inadequate oxygen (O_2_) delivery to the exercising skeletal muscles sets the upper limit of endurance performance in healthy individuals (Bassett and Howley, 2000). In some circumstances (for instance, when O_2_ availability is drastically reduced), however, the central nervous system (CNS) may hold a pivotal role as the ultimate site of exercise termination: the theory of a “central governor” emerged (Noakes, 1997; Noakes et al., 2001). In this context, studies exploring the origin of fatigue along the motor pathway (Gandevia, 2001) provided convincing evidence that cerebral hypoxia may be associated with impaired voluntary activation of the appendicular muscles, i.e., central fatigue (Verges et al., 2012; Goodall et al., 2014a). In fact, appropriate O_2_ delivery to the brain during exercise is critically dependent on both cerebral blood flow and arterial O_2_ content (Smith and Ainslie, 2017). From a clinical standpoint, compromised O_2_ delivery during exercise, secondary to perfusion and/or diffusion abnormalities, is a common denominator across cardiorespiratory diseases (Oliveira et al., 2015). Moreover, chronic exposure to hypoxia, as found in some respiratory disorders, may have structural and functional consequences on the brain (Macey et al., 2008; Li and Fei, 2013) with potential implications for exercise tolerance. Given these premises, and recent advances in non-invasive techniques to interrogate the CNS on exertion [e.g., brain imaging (Perrey, 2008) and stimulations applied along the motor pathway (Millet et al., 2011), particularly on the cortex (Sidhu et al., 2009)], there has been a recent interest on the “brain” response to exercise in patients with chronic cardiorespiratory diseases. The present review, therefore, provides an up-to-date outline of the available literature supporting the contribution of the “brain” in limiting the tolerance to physical exertion in these patients. To reach this overarching goal, the present manuscript addresses the following topics: *(i) A primer on the assessment of neuromuscular function and cerebral hemodynamics in exercise studies; (ii) Can the brain be a limiting factor of exercise tolerance in cardiorespiratory diseases?; and (iii) Summative evidence and research perspectives*.

## A Primer on The Assessment of Neuromuscular Function and Cerebral Hemodynamics in Exercise Studies

### Beyond the Cardiopulmonary Axis: A Rationale for Investigating Fatigue

Patients with chronic cardiorespiratory disorders typically show a reduced capacity to tolerate physical exertion in daily life (Molgat-Seon et al., 2019; Neder et al., 2019b). Cardiopulmonary exercise testing offers an overview of the integrative response to exercise, potentially exposing cardiocirculatory, mechanical-ventilatory and gas exchange abnormalities characteristically observed in these patients (O’Donnell et al., 2019). However, some patients may present with additional functional (e.g., peripheral muscle) abnormalities that may also limit their tolerance to exercise (Chien et al., 2010; Panagiotou et al., 2016; Marillier et al., 2020b). It follows that limitation to physical exertion may arise beyond the cardiopulmonary axis in clinical populations: investigating the underlying mechanisms using various testing modalities (e.g., fresh *vs.* fatigued state, whole-body *vs* single-joint exercise, voluntary *vs.* evoked muscle contractions) might provide important additive information to cardiopulmonary exercise testing.

Innovative theories recently (roughly starting in the late 1990s) challenged the traditional physiological model supporting the notion that limited O_2_ delivery by the cardiocirculatory system to the skeletal muscles is the single mechanism restraining human tolerance to whole-body exercise (Bassett and Howley, 2000). It has been alternatively proposed (Noakes’ “central governor” theory) that skeletal muscle contractile activity may be ultimately regulated by a series of central and peripheral adaptations that prevent the development of organ damage during exercise in both health and disease (Noakes, 1997). Other models have flourished to question, or complement, this theory but all coincide in admitting a core role for central mechanisms in regulating human performance (Marcora, 2008; Amann, 2011; Millet, 2011).

In this context, it is instructive to consider that human performance may be limited by fatigue, defined as a complex (and sometimes disabling) symptom arising from interrelated physiological and psychological underpinnings (Enoka and Duchateau, 2016). Fatigue as a trait (i.e., the amount of fatigue at rest one experience over a given period of time) can be appreciated by self-report; moreover, fatigue can be assessed as a state, e.g., the amount of fatigue one experience during a given task (Gruet, 2018). Exercise-induced fatigue (which may also be referred to as “fatigability”, the extent of fatigue induced by a given amount of work) holds implications for various settings, including those germane to patients with chronic respiratory diseases (Gruet, 2018). Fatigue caused by exercise may be evidenced by any decrease in the ability to exert force or power by a muscle or a muscle group (Bigland-Ritchie et al., 1978), typically referred as “neuromuscular fatigue” (Millet et al., 2011). For the purpose of this concise review, therefore, we will focus on the objective assessments of fatigue as related to muscle performance and neuromuscular alterations.

### Uncovering the Etiology of Fatigue

Neuromuscular fatigue is usually separated into two components (Westerblad and Allen, 2002): *peripheral* and *central*, referring to alterations distal and proximal to the neuromuscular junction, respectively (Edwards et al., 1977). Central alterations, therefore, reflect any changes within the brain and/or in the upper and lower motoneurons (Gandevia, 2001). Of note, both peripheral and central factors are implicated in the development of fatigue, while these two components are thought to be interrelated: motoneuronal recruitment depends on the descending drive from supraspinal sites in the brain while central drive is influenced, in particular, by excitatory and inhibitory muscle afferents (Amann, 2011). Volitional and non-volitional techniques can be used to evaluate neuromuscular fatigue. The two main volitional techniques used in clinical settings to assess muscle strength are: (a) the one-repetition maximum (the greatest weight one can move once over the full range of motion); and (b) the isometric maximal muscle strength (Marillier et al., 2021c). Although a drop in maximal muscle strength during or following a given physical task suggests neuromuscular fatigue, such method may be misleading: it requires optimal subject cooperation and, consequently, unintended submaximal effort may lead to low force production thereby confounding fatigue measurements. Moreover, volitional muscle force assessment cannot differentiate peripheral from central mechanisms of fatigue. To overcome such methodological pitfalls, various types of stimulation at different levels of the neuromuscular pathway can be used to artificially stimulate a muscle or a muscle group (Millet et al., 2011).

**Figure 1:**
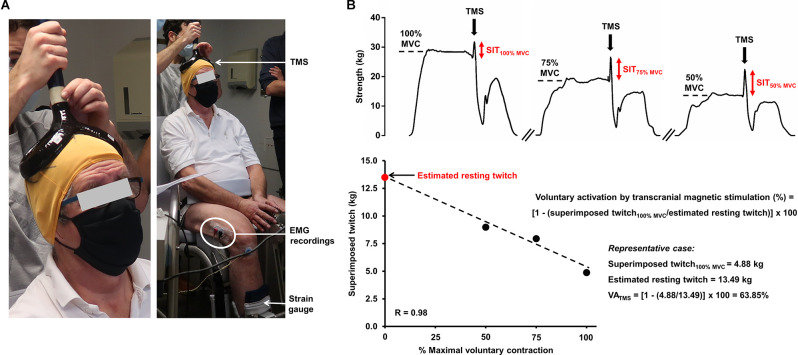
Experimental setting required for the assessment of neuromuscular fatigue using transcranial magnetic stimulation (panel **A**) and determination of voluntary activation by transcranial magnetic stimulation (panel **B**). **(A)** A pulse is delivered to the brain area corresonding to the motor cortex (preliminarily determined) during a voluntary contraction and the evoked muscle strength is recorded with an appropriate ergometer (strain gauge). Strength measures may also be couple with EMG recordings. **(B)** The exposed case depicts the determination of voluntary activation of the (unfatigued) knee extensors by transcranial magnetic stimulation in a 67-year old man with obstructive sleep apnea (apnea-hypopnea index = 35 events/hour). Specifically, the participant are asked to perform a brief maximal voluntary contraction followed by two submaximal contractions at 75% and 50% of maximal voluntary contraction. A pulse is applied to the skull, corresponding to the motor cortex, at these different strength levels after obtaining a plateau. The linear relationship between the voluntary strength and the evoked muscle strength by the pulse (superimposed twitch) allows the estimated resting twitch to be determined. The superimposed twitch obtained at 100% of maximal voluntary contraction and the estimated resting twitch (theoretically obtained on the relaxed muscles) are then used for determining voluntary activation. The representative case shows a clear deficit in cortical voluntary activation in a fresh state since a submaximal value is obtained. Abbreviations. EMG, electromyography; MVC, maximal voluntary contraction; SIT, superimposed twitch; TMS, transcranial magnetic stimulation; VA, voluntary activation.

Assessing peripheral and/or central fatigue can be carried out using electrical and/or magnetic stimulation (Millet et al., 2011; Gruet et al., 2013), providing important insights into the presence and etiology of fatigue. *Peripheral fatigue* can be objectively evidenced by an exercise-induced reduction in muscle strength, evoked by the stimulation of the relaxed muscle either applied to the trunk of the motoneurons or the muscle itself. More specifically, a ~15% drop in isometric quadriceps muscle strength evoked by the stimulation of the femoral nerve after exercise (e.g., cycling or a multi-component exercise training session) is useful to expose significant, and clinically-relevant, peripheral fatigue (Kufel et al., 2002; Burtin et al., 2012). A well-accepted approach to quantify voluntary activation (and, therefore, to appreciate *central fatigue*) is the interpolated twitch technique (Merton, 1954). During a maximal voluntary contraction, the transcutaneous stimulation of a peripheral nerve may elicit additional force if the lower motoneurons have not been fully recruited, i.e., a superimposed twitch emerges. The superimposed twitch amplitude is then compared to the response obtained in the relaxed muscles to quantify voluntary activation (Allen et al., 1995; for further elaboration, see Marillier et al., 2021c). A drop in voluntary activation, during or following exercise, indicates a fall in the descending drive either at the spinal or supraspinal level, i.e., central fatigue. Further insights into the mechanisms of central fatigue can be provided by stimulations applied at the cerebral or spinal levels (Gruet et al., 2013). In that respect, transcranial magnetic stimulation is a complementary, non-invasive technique in which a magnetic pulse is applied to the motor cortex. Voluntary activation quantified by transcranial magnetic stimulation helps uncover supraspinal fatigue, i.e., impaired drive from the motor cortex, a subset of central fatigue (Todd et al., 2003). Figure 1 depicts an overview of the experimental setting required for assessing neuromuscular fatigue by transcranial magnetic stimulation, and subsequent determination of cortical voluntary activation. Specifically, the measure of voluntary activation by transcranial magnetic stimulation relies on pulses applied to the skull (motor cortex) at different levels of voluntary strength. The linear relationship between evoked muscle strength by pulses (i.e., superimposed twitches) obtained at maximal and submaximal levels of voluntary strength allows the estimated resting twitch (i.e., theoretically obtained on the relaxed muscles) to be determined. Alike the interpolated twitch technique, the superimposed twitch obtained at 100% of maximal voluntary contraction and the estimated resting twitch are then used for assessing cortical voluntary activation. Transcranial magnetic stimulation may also offer insights into additional parameters as related to corticospinal excitability and inhibition, such as motor-evoked potentials or cortical silent periods (summarized in Table 1).

**Table 1:** Main parameters assessed by peripheral nerve stimulation and transcranial magnetic stimulation to evaluate muscle fatigue or exercise-induced alterations in peripheral and corticospinal excitability and inhibition.

Technique		Variable	Parameter assessed	Interpretation as related to exercise
Peripheral nerve stimulation		Mmax	Sarcolemmal excitability	↓ amplitude: ↓ sarcolemmal excitability
		Resting twitch	Peripheral muscle contractility	↓ amplitude: peripheral fatigue
		Peripheral VA	Maximal voluntary activation	↓ level: central fatigue
Transcranial magnetic stimulation	Single-pulse	MEP	Corticospinal excitability	↓ amplitude: ↓ corticospinal excitability
		CSP	Duration of intracortical GABA_B_-mediated inhibition	↑ duration: ↑ intracortical inhibition
		SIT	Supraspinal deficit	↑ amplitude: ↑ supraspinal deficit
		Supraspinal VA	Supraspinal voluntary activation	↓ level: supraspinal fatigue
	Paired-pulse	SICI	Magnitude of intracortical GABA_A_-mediated inhibition	↓ conditioning-to-test stimuli ratio: ↑ intracortical inhibition
		LICI	Magnitude of intracortical GABA_B_-mediated inhibition	↓ conditioning-to-test stimuli ratio: ↑ intracortical inhibition

*CSP, cortical silent period; GABA, gamma-aminobutyric acid; LICI, long-interval intracortical inhibition; MEP, motor-evoked potential; Mmax, maximal muscle compound action potential; SICI, short-interval intracortical inhibition; SIT, superimposed twitch; VA, voluntary activation. Exercise-induced reduction (from baseline) in the resting twitch, voluntary activation assessed by peripheral nerve stimulation and voluntary activation assessed by transcranial magnetic stimulation respectively indicate peripheral, central and supraspinal fatigue (for further elaboration, see Investigating the brain on exertion: theoretical framework and key definitions)*.

### On the Complexities of Assessing Neuromuscular Fatigue in Response to Whole-Body Exercise

The assessment of neuromuscular fatigue (its central component in particular) during whole-body exercise is subject to relevant limitation. In fact, exercise-induced corticospinal changes (including central and supraspinal fatigue) may recover quickly (within 2 min) after exercise (Gruet et al., 2014). The net result may be an underestimation of the potential corticospinal alterations if there is a sizable delay between exercise cessation and fatigue assessment. For instance, we were unable to transfer patients with moderate-to-severe fibrotic interstitial lung disease (*f*-ILD) after constant-load cycling to a dedicated ergometer in a timeframe shorter than 3 min (Marillier et al., 2021a). Moreover, time-related changes in the parameters reflecting central fatigue cannot be obtained during whole-body exercise as measurements are restricted to the post-exercise period (either after exhaustion or after a predetermined exercise duration has elapsed). Also important, patients may not tolerate exercise intensities sufficiently high to induce appreciable neuromuscular fatigue, as a strategy to cope with exertional symptoms, particularly those primarily limited by breathlessness or angina (Guthrie et al., 2001; Lemmens et al., 2008; Gardner et al., 2011; van Buul et al., 2017). Hence, assessing fatigue at a single time point equivalent to symptom limitation may not reflect the usual level of fatigue faced by patients in daily life. Although not widely available, Doyle-Baker and colleagues (Doyle-Baker et al., 2018) recently developed a new ergometer enabling a switch from recumbent cycling to isometric set-up to assess neuromuscular fatigue within 1 s. This may allow measurements at any moment during submaximal exercise intensities (to appreciate time-related changes in corticospinal parameters) or post-exercise fatigue measurements without substantial delay during whole-body (cycling) exercise. Such an innovative ergometer may prove particularly useful to advance the understanding of fatigue faced by severely-limited patients with cardiorespiratory disorders during dynamic exercise.

### Single-Joint Exercise: A Valuable Alternative to Assess Neuromuscular Fatigue

Single-joint exercise, involving an endurance test from a specific muscle group, might also prove useful to investigate neuromuscular fatigue, particularly in severely-limited patients. Owing to the small muscle mass involved, it only elicits mild increases in ventilation and allows a focused investigation of muscle function (Gruet, 2018), which is highly relevant when assessing patients limited by mechanical-ventilatory abnormalities during whole-body exercise (Marillier et al., 2020a). Interrogating lower limb muscle function and neuromuscular fatigue might prove particularly relevant as they are the most impaired muscles in chronic respiratory disorders (Maltais et al., 2014; Gruet et al., 2017). Due to the limited experimental set-up (Figure 1), protocols involving isometric contractions are easily implementable, providing reliable estimates of limb muscle strength and endurance in both healthy and clinical populations (Bachasson et al., 2013; Machado Rodrigues et al., 2017). Contractions are performed at an intensity relative to predetermined maximal voluntary strength to compensate for a (likely) lower muscle strength in patients, so as not to influence findings relating to neuromuscular fatigue. Such methodological feature is important to avoid the confounding effects of low muscle mass and strength usually found in patients with chronic cardiorespiratory disorders (Buller et al., 1991; Holland, 2010; Maltais et al., 2014). Of note, time delay between task failure and (central) fatigue assessment is not a relevant issue to (isometric) single-joint exercise. In fact, exercise-induced corticospinal alterations (of whom central and supraspinal fatigue) may be investigated all over the course of the task of interest (e.g., after a set of contractions; Marillier et al., 2018a), being an important practical advantage of such testing modality.

### Cerebral Hemodynamics: A Complementary Outcome to Fatigue Assessment

**Figure 2:**
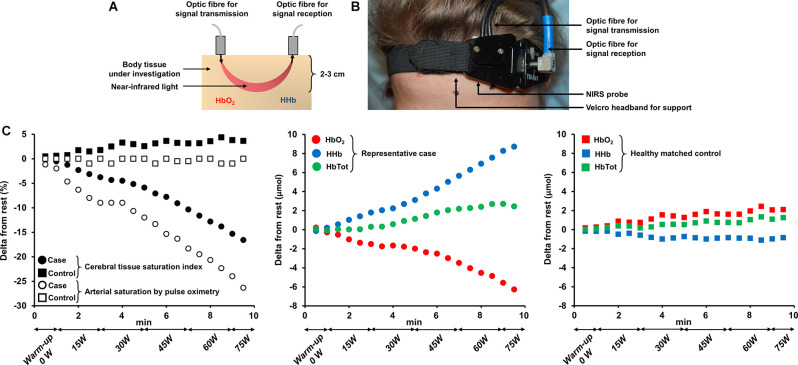
Principles of near-infrared spectroscopy technology (panel **A**), experimental setting for assessing cerebral oxygenation at the prefrontal cortex (panel **B**) and time-course changes in prefrontal cortex hemodynamics in a representative patient with severe activity-related hypoxemia and a healthy matched control (panel **C**). **(A,B)** Near-infrared light illuminates the tissue under investigation and is reflected back to the receptors. The technique allows the determination of the concentration of two main chromophores: oxy- and deoxyhemoglobin. Near-infrared spectroscopy is typically suitable for the investigation of cerebral hemodynamics in structures no deeper than approximately 2–3 cm below the surface of the skull e.g., the prefrontal cortex. **(C)** The exposed case depicts a substantial drop in cerebral tissue saturation index by near-infrared spectroscopy due to severe hypoxemia in a 64-year old woman with smoking-related interstitial lung disease (total lung capacity = 67% predicted, lung diffusing capacity for carbon monoxide = 39% predicted) during an incremental exercise test to symptom limitation on a cycle ergometer. Time-course changes in oxy-, deoxy- and total hemoglobin (calculated as the sum of oxy- and deoxyhemoglobin) concentrations are also depicted. Note the primary complaint of dizziness on exertion for this patient. Corresponding measurements obtained in a healthy matched (for sex and age) control over the same timeframe are also available for comparison. Abbreviations. HbO_2_, oxyhemoglobin; HHb, deoxyhemoglobin; HbTot, total hemoglobin.

The assessment of cerebral hemodynamics is a complementary strategy to get valuable insights into the “brain” response to exercise. Near-infrared spectroscopy (NIRS) is particularly suitable to investigate cerebral tissue oxygenation and hemodynamics during exercise (Perrey, 2008). As a non-invasive method, it can be used repeatedly or over prolonged durations. It is also relatively easy to handle when the experimenter is trained, and portable (especially recently developed wireless systems). NIRS has an acceptable signal-to-noise ratio even when the subject is in motion (Perrey, 2008) such as during whole-body exercise (though a better signal is usually obtained on cycling compared to treadmill). The principles of NIRS and the experimental setting for the assessment of cerebral oxygenation are shown in Figure 2. NIRS allows the determination of the concentration of two main chromophores (oxy- and deoxyhemoglobin), total hemoglobin (calculated as the sum of oxy- and deoxyhemoglobin) and an estimate of tissue O_2_ saturation (Ferrari et al., 2004; Ferrari and Quaresima, 2012). This optical technology relies on the use of infrared wavelength light (650–950 nm). Photons in the near-infrared spectrum are able to cross tissue over several centimeters, within the cerebral cortex in particular (Perrey, 2008). Near-infrared light illuminates the body tissue under investigation and is reflected back to the receptors. In this context, the difference between the intensity of the light emitted and received provides information on the concentration of the different chromophores within a tissue at a given wavelength. Thus, when the intensity of the light received at the receptors is low, the absorption of infrared photons is high and therefore indicates that the concentration of the chromophore in question is also high (Wahr et al., 1996). The technique offers an excellent temporal resolution (high sampling frequency), allowing continuous and precise estimations of changes in cerebral hemodynamics induced by exercise (Huppert et al., 2006). However, its spatial resolution remains low, and NIRS only interrogates structures within 2–3 cm below the surface of the skull, e.g., the motor or prefrontal cortex (Perrey, 2008). In addition, NIRS may be used to assess the functional connectivity across cortical areas (Xu et al., 2017; Urquhart et al., 2019), shedding light on specific functional architecture during fatiguing motor tasks among clinical populations (Rhee and Mehta, 2018). Cerebral blood flow might also be obtained by NIRS, but it requires the injection of the light-absorbing tracer indocyanine green (Kuebler et al., 1998). Transcranial Doppler ultrasound can also be used to assess cerebral blood flow during exercise (Smith and Ainslie, 2017): data obtained in cardiorespiratory diseases [chronic heart failure (CHF) and chronic obstructive pulmonary disease (COPD)] are herein presented when relevant to the scope of this review. Other imaging techniques, such as magnetic resonance imaging, however, remain poorly implementable during exercise (Perrey, 2008).

### The “Healthy” Brain: Evidence of Its Contribution to Exercise Limitation

Skeletal muscles are exquisitely sensitive to any impairment in O_2_ delivery, resulting in a disturbance of muscle metabolism (Adams and Welch, 1980). Metabolites involved in the development of peripheral fatigue [e.g., H^+^ or inorganic phosphate (Allen et al., 2008)] accumulate faster when muscle O_2_ supply is reduced (Hogan et al., 1999), eventually leading to impaired muscle contractility (Amann and Calbet, 2008). In this context, exaggerated muscle fatigability (due to compromised muscle O_2_ delivery) may stimulate group III/IV muscle afferents to inhibit the descending central drive (Amann et al., 2009), acting as a protective mechanism to avoid tissue injury but reducing exercise performance under hypoxic conditions (Amann et al., 2006). Yet, the severity of hypoxemia plays a pivotal role in the respective contribution of peripheral and central fatigue in limiting the tolerance to exercise in healthy individuals (Goodall et al., 2014a). Compromised cerebral oxygenation is a consistent finding in healthy individuals exercising in hypoxia (Subudhi et al., 2009; Marillier et al., 2021d). Although some studies reported an increase in cerebral blood flow to compensate for a reduced arterial O_2_ content (Subudhi et al., 2009; Rasmussen et al., 2010; Goodall et al., 2014b), cerebral O_2_ delivery and oxygenation during exercise in hypoxia were found to be reduced (Goodall et al., 2012, 2014b). The independent (of peripheral fatigue) inhibition of the central drive, due to cerebral hypoxia, was demonstrated by Amann, Romer (Amann et al., 2007). In this study, participants performed a constant-load cycling test to exhaustion under normoxia, moderate and severe hypoxemia (end-exercise arterial saturation by pulse oximetry, SpO_2_ = 94%, 82% and 67%, respectively). A low central motor output (integrated EMG) was observed in line with a critical drop in cerebral oxygenation in the latter condition. In contrast, supplemental O_2_ reversed hypoxemia and exerted positive consequences on cerebral oxygenation and central motor output, leading to a prolonged exercise duration (171 ± 61%) only under severe hypoxemia. The authors therefore provided convincing evidence in favor of a progressive switch from peripheral to central fatigue as cerebral hypoxia worsened (Amann et al., 2007), a result confirmed in response to single-joint exercise under severe hypoxemia (Goodall et al., 2010). Although some chronic cardiorespiratory conditions are not necessarily associated with severe (acute) exertional hypoxemia, these results provide a rationale for investigating the consequences of compromised O_2_ delivery to the CNS in clinical populations. Chronic (intermittent) hypoxia may also be a relevant feature in these patients with potential deleterious consequences on the brain structure and function (Li and Fei, 2013; Rosenzweig et al., 2015).

## Can The Brain Be A Limiting Factor of Exercise Tolerance in Cardiorespiratory Diseases?

### Chronic Heart Failure

Exercise intolerance in patients with CHF typically stems from two major pathophysiological mechanisms: (i) impaired cardiac output, and (ii) increased sympathetic activity. The former mechanism results in a low O_2_ delivery to the exercising muscles while the latter leads to increased respiratory neural drive and, consequently, greater ventilation and dyspnea at a given exercise intensity (Plachi et al., 2020). A putative influence of impaired cerebral blood flow and oxygenation in reducing the tolerance to whole-body exercise tolerance in CHF has been put forward by several authors (Koike et al., 2006; Rooks et al., 2010; Fu et al., 2011). For instance, Fu et al. found that while cerebral oxygenation and perfusion (prefrontal cortex oxy- and total hemoglobin by NIRS) increased throughout exercise in healthy controls and in CHF patients (New York Heart Association class II), they decreased at peak exercise in more severe (class III) patients (Fu et al., 2011). In fact, larger cerebral perfusion at peak exercise independently predicted peak O_2_ uptake, the latter being also lower in class III patients. Decrements in cerebral oxygenation in CHF may be secondary to the combining effects of impaired cardiac output, and an excessive ventilatory response to exertion leading to a low arterial partial pressure of CO_2_—a potent cerebral vasoconstrictor (Koike et al., 2004). Whether increased sympathetic nervous activity, a key feature of CHF, is beneficial or detrimental for the regulation of cerebral blood flow (due to cerebral vasoconstriction) during exercise remains to be determined (Brassard and Gustafsson, 2016). A comprehensive investigation revealed abnormally low resting internal carotid artery blood flow and middle/posterior cerebral artery velocities (Doppler ultrasound) in CHF (Smith et al., 2019). These abnormalities were further magnified during exercise: compared to resting conditions, only middle cerebral artery velocity increased in patients with CHF while flow/velocity increased in the three arteries in controls (Smith et al., 2019). In this context, the contribution of these impairments in exacerbating central fatigue and, consequently, reducing exercise tolerance in patients with CHF remains elusive at this point in time, although Brassard and Gustafsson (Brassard and Gustafsson, 2016) recently suggested that impaired brain perfusion and oxygenation may be relevant players to exercise intolerance in this patient population. Hopkinson and colleagues (Hopkinson et al., 2013) investigated the impact of CHF on the neuromuscular function using peripheral and transcranial magnetic stimulation before and after incremental cycling to symptom limitation. These authors reported greater quadriceps muscle susceptibility to fatigue in patients vs. controls, but voluntary activation was not altered by exercise in both groups, i.e., central fatigue was not accentuated by the disease. Yet, given the fact that neuromuscular assessments were performed 10 min after cycling, any central fatigue may have been overlooked in this study since it typically recovers within 2 min after exercise (Gruet et al., 2014).

### Chronic Obstructive Pulmonary Disease

**Figure 3:**
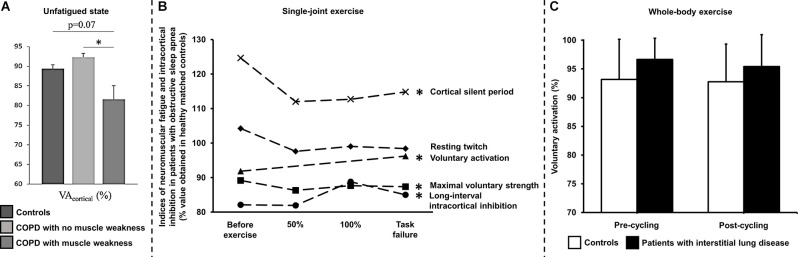
Assessment of neuromuscular fatigue by peripheral nerve and transcranial magnetic stimulations in chronic cardiorespiratory disorders using various exercise modalities. **(A)** Deficits in voluntary activation of the unfatigued (i.e., with no prior fatiguing exercise) knee extensors by transcranial magnetic stimulation has been demonstrated in patients with chronic obstructive pulmonary disease (COPD) showing peripheral muscle weakness in comparison to their counterparts with no muscle weakness. *p < 0.05: patients with vs. with no peripheral muscle weakness. Reproduced and modified, with the permission of the publisher from: Alexandre et al. (2020). **(B)** Lower maximal voluntary strength and voluntary activation by transcranial magnetic stimulation of the knee extensors but similar resting twitch have been shown in patients with severe obstructive sleep apnea syndrome throughout single-joint exercise (repeated knee extensions to task failure, starting at 35% of maximal voluntary strength) in comparison to healthy controls. Greater magnitude (long-interval intra-cortical inhibition) and duration (cortical silent period) of intra-cortical inhibition have also been reported using transcranial magnetic stimulation in these patients compared to their healthy counterparts. *Indicates a significant difference for a given parameter throughout exercise between patients with obstructive sleep apnea using healthy controls as a reference (*p* < 0.05). Data obtained before and after treatment by continuous positive airway pressure were pooled since the intervention did not improve cortical impairments in patients. 50% and 100% refer to data obtained at 50% and 100% of the duration of the shortest test i.e., before or after treatment by continuous positive airway pressure. Reproduced and modified, with the permission of the publisher from: Marillier et al. (2018a). **(C)** Voluntary activation measured using the twitch interpolation technique in response to magnetic stimulation of the femoral nerve in controls and in patients with fibrotic interstitial lung disease before and after a constant-load (60% peak work rate) exercise test to symptom limitation under medical air. Voluntary activation did not significantly differ between controls and patients, being unaltered by exercise in both groups, despite severe exertional hypoxemia in patients. Voluntary activation was assessed ~3 min after whole-body exercise in these subjects. Time delay between exercise cessation and fatigue assessment is a limitation to capture central fatigue after whole-body exercise. Similar observations have been made in patients with heart failure (Hopkinson et al., 2013). Reproduced and modified, with the permission of the publisher from: Marillier et al. (2021a).

Exercise limitation in COPD is multifactorial (Neder et al., 2019a) and includes impairment in gas exchange, respiratory-mechanical and cardio-circulatory abnormalities, and, in some patients, skeletal muscle dysfunction (Marillier et al., 2020b). Using magnetic stimulation of the femoral nerve, Vivodtzev et al. (2008) reported a lower voluntary activation of the quadriceps in patients with advanced COPD compared to controls. The authors imputed these results to the deleterious consequences of severe deconditioning and/or chronic hypoxemia on the CNS. Of note, changes in voluntary activation correlated with improvement in quadriceps maximal strength after 12 weeks of endurance training (Vivodtzev et al., 2008). In the same vein, Alexandre et al. (2014) demonstrated a lower quadriceps muscle strength in the context of reduced cortical activity (prefrontal, premotor and primary motor areas) in COPD vs. controls despite the lack of overt hypoxemia during wakefulness. Yet, non-hypoxemic patients with COPD during wakefulness may present with repeated episodes of arterial desaturation during sleep (Levi-Valensi et al., 1992). In this context, the same group (Alexandre et al., 2016) revealed that patients facing nocturnal hypoxemia showed higher circulating levels of S100 calcium-binding protein B, a marker of brain injury; moreover, they reported lower supraspinal activation in response to transcranial magnetic stimulation during a maximal voluntary contraction of the quadriceps in “desaturators” vs. “non-desaturators”. Recent findings from these authors (Alexandre et al., 2020) also indicate a deficit in voluntary activation from the motor cortex in COPD patients with peripheral muscle weakness (Figure 3). Collectively, these data support a contribution of central and/or supraspinal abnormalities to peripheral muscle weakness in COPD, particularly in patients regularly exposed to hypoxia.

Exertional hypoxemia has also been shown to compromise cerebral oxygenation response to whole-body exercise in COPD: “desaturators” (peak exercise SpO_2_ ~86%) had minimal increase in prefrontal cortex oxyhemoglobin by NIRS in comparison to “non-desaturators” (Oliveira et al., 2012). Supplemental O_2_ (fraction of inspired O_2_ = 0.4) improved cerebral oxygenation and exercise capacity only in “desaturators” (Oliveira et al., 2012). Greater cerebral blood flow, however, may compensate for a lower arterial O_2_ content, maintaining local O_2_ delivery in these patients (Vogiatzis et al., 2013; Hartmann et al., 2014). The potential neuromuscular consequences of compromised cerebral oxygenation/O_2_ delivery during whole-body exercise in COPD remain to be investigated.

### Chronic Obstructive Pulmonary Disease/Heart Failure Overlap

**Figure 4:**
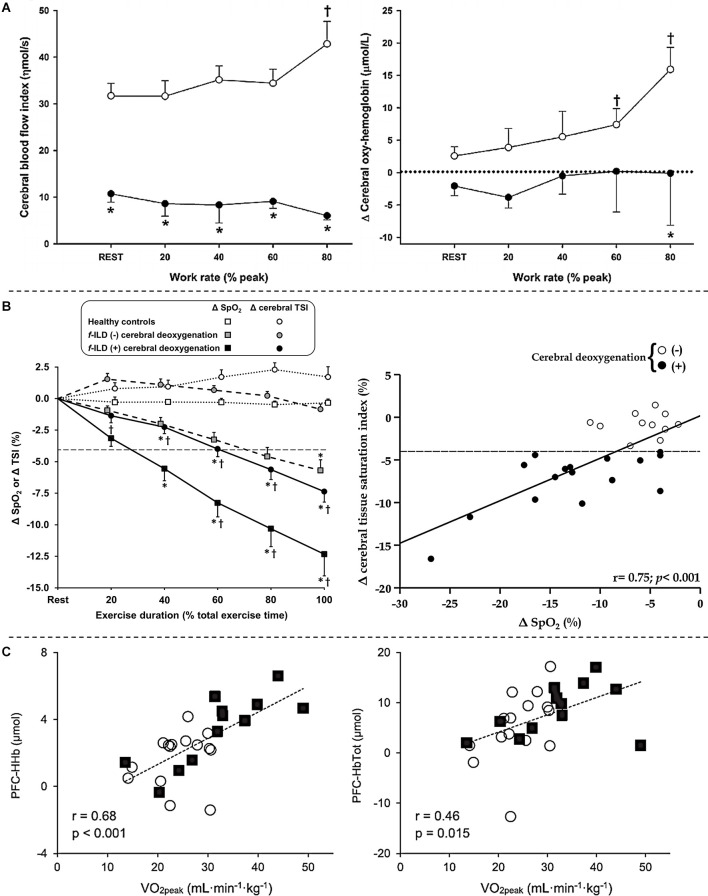
Cerebral hemodynamics assessed by near-infrared spectroscopy in patients with chronic obstructive pulmonary disease-heart failure overlap (panel **A**), fibrotic interstitial lung disease (panel **B**) and severe obstructive sleep apnea (panel **C**) during whole-body exercise. **(A)** Lower mean arterial pressure and cardiac output together with low levels of arterial partial pressure of CO_2_ in patients with overlapping diseases led to poorer prefrontal cerebral blood flow (*left*) and oxygenation (*right*) in comparison to isolated chronic obstructive pulmonary disease during a 4-stage (4 min each at 20% to 80% of peak work rate with 4 min rest in between) cycle ergometer test. Open and closed symbols depict patients with isolated chronic obstructive pulmonary disease and overlapping diseases, respectively. *Between-group differences at a given time point; ^†^intra-group differences vs. rest. Reproduced and modified, with the permission of the publisher from: Oliveira et al. (2016). **(B)** Profound exertional hypoxemia, a key feature of interstitial lung disease, led to poorer cerebral oxygenation compared to healthy controls (*left*) and impairs cerebral oxygenation in a dose-dependent fashion in these patients (*right*) during incremental exercise testing on a bicycle ergometer. Significant cerebral deoxygenation in patients was defined as a ≥4% drop (dashed line) in cerebral tissue saturation index (TSI) as none of the 12 healthy matched subjects showed a decrease above 3% at the peak of incremental cycling in this study. *f*-ILD (-) and *f*-ILD (+) therefore refer to patients with fibrotic interstitial lung disease who presented with or without significant cerebral deoxygenation, respectively. Data are presented as quintiles of total exercise duration (*left*). The relationship between changes (Δ) in TSI and O_2_ saturation by pulse oximetry (SpO_2_) is from rest to peak exercise in patients (*right*). *p < 0.05: patients with *f*-ILD vs. healthy controls; ^†^*p* < 0.05: patients with *f*-ILD showing vs. showing no cerebral deoxygenation for Δ SpO_2_ or Δ TSI at a specific exercise time. Reproduced and modified, with the permission of the publisher from: Marillier et al. (2021b). **(C)** Changes from rest to peak exercise in prefrontal cortex deoxy- (*left*) and total (*right*) hemoglobin during an incremental exercise test in patients with obstructive sleep apnea (open circles) and healthy matched controls (filled squares) correlated with peak O_2_ uptake. Reproduced and modified, with the permission of the publisher from: Marillier et al. (2018b).

**Figure 5:**
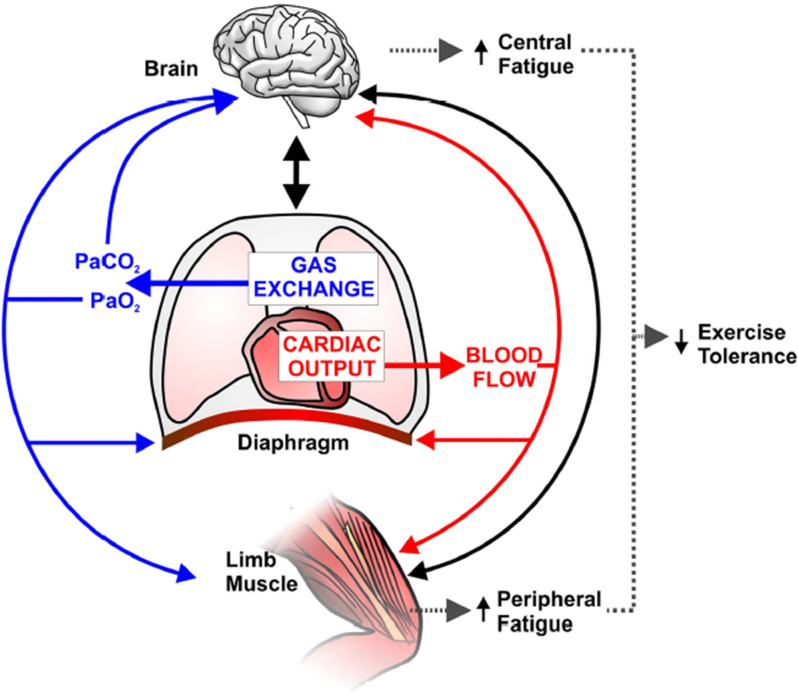
Schematic representation of potential implications of abnormal pulmonary gas exchange and central hemodynamics on central nervous system (brain) and peripheral skeletal muscle function during exercise in combined chronic obstructive pulmonary disease-heart failure. Compromised O_2_ delivery to the brain and active limb muscles can occur as a consequence of impairments in gas exchange (e.g., a reduction in arterial O_2_ pressure; PaO_2_) and/or decreased cardiac output and thus blood flow. A large fraction of an already reduced cardiac output can be directed to the overloaded respiratory muscles (due to increased work of breathing), therefore further decreasing active limb muscle perfusion and accentuating peripheral fatigue. *Solid blacklines* indicate that increased afferent information from the respiratory and peripheral muscles and/or impaired cerebral oxygenation may decrease motor drive (i.e., central fatigue). In this context, it is conceivable that central and peripheral fatigue potentiate each other and contribute to early exercise cessation in coexistent COPD-CHF. Reproduced and modified, with the permission of the publisher from: Oliveira et al. (2015).

As already mentioned, impaired O_2_ delivery is a common key pathological mechanism of cardiorespiratory diseases such as COPD and CHF (Oliveira et al., 2015). Unfortunately, these two conditions coexist in up to a third of elderly patients with a primary diagnosis of either disease (Rutten et al., 2005). The combined effects of “hypoxic” (COPD-induced) and “ischemic” (CHF-related) hypoxia may impair cerebral blood flow and oxygenation during whole-body exercise in coexistent COPD-CHF. In fact, it was found that lower mean arterial pressure and cardiac output in association with a low arterial partial pressure of CO_2_ in COPD-CHF led to poorer resting cerebral blood flow and oxygenation vs. COPD (Figure 4; Oliveira et al., 2013, 2016). Such differences were magnified when transitioning to exercise: whereas cerebral blood flow increased by ~40% in COPD, a drop of ~10% was observed in COPD-CHF (Oliveira et al., 2016). It remains unknown whether impaired cerebral O_2_ delivery translates into exaggerated manifestations of central fatigue in patients with COPD-CHF. The negative cardiopulmonary interactions which may precipitate neuromuscular fatigue in COPD-CHF are summarized in Figure 5.

### Interstitial Lung Disease

Fibrotic interstitial lung disease constitutes a group of disorders from diverse aetiologies, characterized by alveolar/interstitial damage associated with variable degrees of inflammation and fibrosis (Travis et al., 2013). Typical findings in *f*-ILD include increased sensations of dyspnea as the primary complaint and poor exercise tolerance (O’Donnell et al., 2017). Exercise limitation in these patients arises from a variety of contributors including impaired pulmonary gas exchange (Agusti et al., 1991), abnormalities in respiratory mechanics (Faisal et al., 2016) and cardio-circulatory abnormalities (Hansen and Wasserman, 1996). Extreme exertional hypoxemia, owing to impaired O_2_ diffusion, alveolar ventilation-capillary perfusion mismatch and microvascular destruction (Hamer, 1964; Agusti et al., 1991) is a key feature of *f*-ILD: O_2_ saturation in the low 70% might be observed in some patients (Du Plessis et al., 2018). However, the influence of such profound hypoxemia on cerebral oxygenation during whole-body exercise and its consequences on exercise tolerance were unknown until recently. In this context, we demonstrated that exercise-related hypoxemia impairs cerebral oxygenation in a dose-dependent fashion in these patients (Figure 4). Moreover, impaired cerebral oxygenation was identified as an independent predictor of poor whole-body exercise capacity (Marillier et al., 2021b). In patients presenting with severe hypoxemia, a marked reduction in cerebral oxygenation may have led to an independent (i.e., from muscle afferents; Goodall et al., 2014a) reduction in central drive. In fact, ~50% of patients with *f*-ILD showing significant cerebral deoxygenation had peak-exercise SpO_2_ ≤80% (Marillier et al., 2021b), a value at which cerebral hypoxia and, consequently, central fatigue are considered as prominent mechanisms reducing exercise tolerance (Goodall et al., 2014a). Due to the methodological limitations inherent to the investigation of neuromuscular fatigue after whole-body exercise, however, we were unable to appropriately test for the presence of central fatigue in these patients (Figure 3; Marillier et al., 2021a).

### Obstructive Sleep Apnea

Obstructive sleep apnea (OSA) syndrome is characterized by repeated episodes of partial or complete pharyngeal collapses during sleep (Levy et al., 2015). Direct consequences of these events include intermittent hypoxia and hypercapnia, sleep fragmentation and increased respiratory efforts, ultimately leading to sympathetic activation, oxidative stress and systemic inflammation (Levy et al., 2015). In addition to increased cardiovascular and metabolic mortality associated with OSA (Marin et al., 2005), there are compelling evidence documenting hemodynamic, structural and functional repercussions on the brain (Rosenzweig et al., 2015). In this context, hypotrophic changes of gray matter have been mainly reported in prefrontal, hippocampal, para-hippocampal, and cerebellar areas while white matter is also likely impacted (Rosenzweig et al., 2015).

In this context, specific structural alterations may have had direct implications on the response to exercise in OSA. For instance, Joo and colleagues (Joo et al., 2013) reported cortical thinning in the pericentral gyri corresponding to the primary motor area in particular, while Macey et al. (2008) suggested an impairment of axons from this cortical area. To our knowledge, only one study had previously reported reduced quadriceps maximal strength and endurance in OSA patients compared to healthy controls (Chien et al., 2010). Still, this study was not in a position of teasing out the origin (peripheral vs. central) of such alterations. We recently documented the neuromuscular consequences of OSA using a comprehensive investigation of the neuromuscular function (femoral nerve electrical stimulation and transcranial magnetic stimulation) during exercise (Marillier et al., 2018a). Repeated knee extensions were used to track neuromuscular alterations throughout exercise. We found that muscle endurance (time to task failure) was ~30% lower in patients while maximal strength of the quadriceps was consistently lower throughout exercise compared to controls. Voluntary activation assessed by femoral nerve and transcranial magnetic stimulation was lower in OSA *vs.* controls, independently of exercise time. In other words, patients with OSA were unable to appropriately activate their muscles, part of this deficit originating from supraspinal (motor cortex) sites (Marillier et al., 2018a). Such findings were found in the presence of longer cortical silent periods and greater long-interval intracortical inhibition in OSA, respectively indicating a greater duration and magnitude of intracortical inhibition vs. controls. Importantly, the kinetics of the potentiated twitch were similar in both groups thereby excluding a potential contribution of peripheral fatigue to the observed functional (strength/endurance) abnormalities (Figure 3). Overall, this study demonstrates that patients with severe OSA have cerebral impairments which are poised to contribute to muscle dysfunction, i.e., reduced knee extensors strength and endurance.

Some results also suggest that resting cerebral hemodynamic abnormalities [e.g., impaired cerebrovascular reactivity (Prilipko et al., 2014) or reduced cerebral blood flow (Yadav et al., 2013; Chen et al., 2017)] in OSA may translate into cardiovascular and ventilatory dysregulations during exercise (Kumar et al., 2012). Given these premises, we investigated whether altered cerebral hemodynamics during whole-body exercise may contribute to an impairment in exercise tolerance in OSA (Marillier et al., 2018b). In addition to a lower peak O_2_ uptake, we found a lower increase (from baseline) at peak exercise in prefrontal cortex deoxy- and total hemoglobin in patients with OSA vs. healthy controls, suggesting an impairment in cerebral O_2_ extraction and cerebral blood flow in the former group (Marillier et al., 2018b). Furthermore, these indices positively correlated with exercise capacity (peak work rate and O_2_ uptake) in our sample (Figure 4). These data lend support to the notion that impaired cerebrovascular response to whole-body exercise may have a contributory role in reducing exercise tolerance in OSA (Marillier et al., 2018b). The latter assertion, however, necessitates further investigation in studies measuring central fatigue in severe OSA.

## Summative Evidence

**Figure 6:**
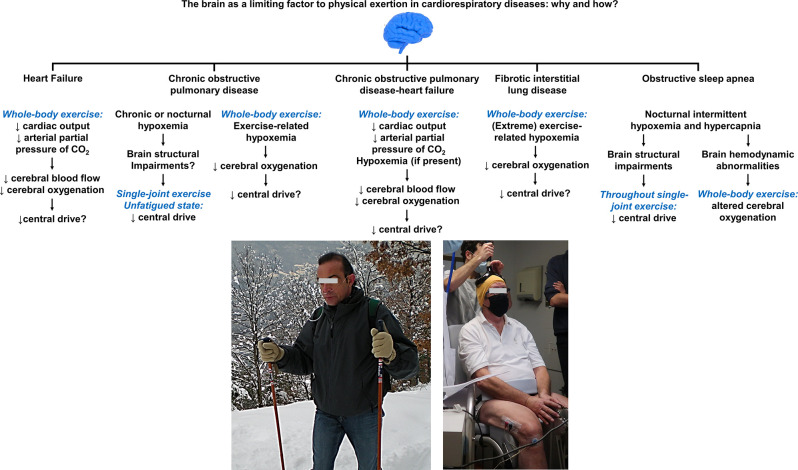
Summary of the mechanisms by which the brain may act as a limiting factor to physical exertion in cardiorespiratory diseases. Compromised cerebral oxygenation during whole-body exercise, owing to poor brain perfusion and/or exercise-related hypoxemia (e.g., a patient with chronic obstructive pulmonary disease requiring supplemental O_2_, ***left picture***), appears as a common denominator in such diseases. Supporting evidence for the deleterious influence of impaired cerebral oxygenation on central/supraspinal fatigue (using transcranial magnetic stimulation, ***right picture***) during exercise are, nevertheless, currently lacking in most of the showcased conditions. Despite deficits in cerebral perfusion during exercise in patients with heart failure, no study to date has established the contribution of central fatigue to limit patient’s ability to sustain prolonged exercise. Deficits in voluntary activation from the motor cortex in unfatigued state have been shown in specific subpopulations of patients with chronic obstructive pulmonary disease, whose implications upon exertion are uncertain. It remains also unknown whether impaired cerebral O_2_ delivery translates into exaggerated manifestations of central fatigue in patients with chronic obstructive pulmonary disease-heart failure. Exertional hypoxemia impairs cerebral oxygenation in a dose-dependent fashion in fibrotic interstitial lung disease. In light of the unparalleled severity of hypoxemia observed in this patient population, cerebral hypoxia likely holds an independent impact on the descending motor drive although this remains to be spelt out. Lower cortical drive to the active muscles during single-joint exercise have been shown in severe obstructive sleep apnea and may contribute to skeletal muscle dysfunction; the repercussions of these findings during whole-body exercise are uncertain although the cerebral hemodynamic response to incremental cycling relates to exercise capacity in these patients. Future mechanistic studies may focus on investigating the potential neuromuscular consequences of the featured (and other hitherto unexplored) cardiorespiratory conditions in whom fatigue is typically a major complaint during daily physical activities.

The present review outlines some limited evidence suggesting a putative role for the brain as a factor limiting the tolerance to physical exertion in patients suffering from major cardiorespiratory diseases. Impairment in cerebral oxygenation during whole-body exercise, secondary to poor brain perfusion and/or exertional hypoxemia, seems an ubiquitous finding in patients with advanced disease (Fu et al., 2011; Oliveira et al., 2012, 2016; Marillier et al., 2021b). Yet, to date, there is only scarce experimental evidence supporting a decisive role for impaired cerebral oxygenation on central/supraspinal fatigue during whole-body exercise in clinical populations (Figure 6). Despite impressive deficits in cerebral perfusion on exercise in patients with moderate-to-severe CHF (Fu et al., 2011; Smith et al., 2019), no study to date has established the contribution of central fatigue to limit patient’s ability to sustain prolonged exercise. Recent studies have reported deficits in voluntary activation from the motor cortex in unfatigued state in some patients with COPD (Vivodtzev et al., 2008; Alexandre et al., 2016, 2020) which might have implications upon exertion. Worse cerebral oxygenation in COPD patients with coexistent CHF (Oliveira et al., 2016) suggests that the perfusion component is likely instrumental to overcome the cerebral auto-regulation mechanisms in patients with combined cardio-respiratory disease. In patients with *f*-ILD, poor cerebral oxygenation has been independently associated with exercise intolerance (Marillier et al., 2021b). Given the severity of exercise-related hypoxemia observed in this population (Du Plessis et al., 2018), it is conceivable that cerebral hypoxia may hold an independent (from sensory muscle afferents) impact on the descending motor drive. Finally, patients with OSA display a lower supraspinal drive during single-joint exercise which likely contribute to skeletal muscle dysfunction (Marillier et al., 2018a). As in other clinical populations, the consequences of these findings to whole-body exercise tolerance remains uncertain.

## Research Perspectives

Central abnormalities on exertion might also be expected in hitherto unexplored respiratory disorders. Cystic fibrosis (CF) shares some common features with COPD such as inflammation and chronic hypoxemia (Gruet et al., 2017); of note, these features might have harmful consequences to the CNS that may contribute to muscle dysfunction in COPD (Vivodtzev et al., 2008; Alexandre et al., 2016). Besides, transmembrane conductance regulator protein, which is defective in CF, is abundantly expressed in brain neurons (Guo et al., 2009) of cortical areas involved in motor control and regulation of neuromuscular fatigue (Tanaka and Watanabe, 2012). Using magnetic stimulation of the femoral nerve throughout an incremental, isometric quadriceps endurance test, Gruet and colleagues (Gruet et al., 2016) examined neuromuscular fatigue in patients with mild-to-moderate CF. Quadriceps muscle endurance and fatigability were comparable in patients and healthy controls, while (central) voluntary activation only tended to be lower at baseline (fresh state) in the former group. Yet, the harmful consequences of the abovementioned parameters (e.g., inflammation) on brain function might be more relevant in patients with more severe genotypes and phenotypes.

Severe asthma is characterized by airway inflammation, hyperresponsiveness, and obstruction. A recent systematic review and meta-analysis reported cognitive impairments in patients with asthma, the severity of the disease being singled as a key modulator of such abnormalities (Irani et al., 2017). Beyond demographic and socioeconomic factors, biological causes (alike in COPD and OSA for instance; Dodd et al., 2010; Rosenzweig et al., 2015) might hold a contributory role for the cognitive dysfunction in severe asthma. In fact, it has been suggested that this might be related to intermittent cerebral hypoxia (Irani et al., 2017). Although symptoms of severe fatigue are highly prevalent in this population (Van Herck et al., 2018), no study to date has assessed whether central fatigue might arise in response to repeated bouts of cerebral hypoxia induced by exertion in severely asthmatic patients.

Once identified, future mechanistic studies may focus on strategies seeking to ameliorate, or reverse, potential abnormalities arising from the CNS in these different chronic diseases. Although we failed to alter supraspinal defects (voluntary activation, intracortical inhibition) and cerebral hemodynamics in patients with severe OSA after 8 weeks of continuous positive airway pressure (CPAP; Marillier et al., 2018a, b), treatment duration might have been insufficient to positively impact on these outcomes. For instance, 12 months of CPAP was required to cause almost complete recovery of brain structural changes in another study (Castronovo et al., 2014). Exercise training is known to have a protective influence on the brain structural and functional integrity in the healthy elderly (Jackson et al., 2016). Physical exercise specifically conferred structural benefits (i.e., greater volume) in the precentral gyrus (motor area) and in the supplementary motor area in elderlies (Erickson et al., 2010); interestingly, these are potentially beneficial changes for neuromuscular functioning relevant to exertion (Alexandre et al., 2014). In fact, a recent investigation reported that a 12-week strength training program led to a significant increase in voluntary activation of the quadriceps in mobility-limited older individuals, which was linked with improvement in gait speed (Hvid et al., 2016). Concerning patients with cardiorespiratory disorders, a study showed that a 12-week endurance training program improved maximal strength and central drive to the quadriceps in hypoxemic, severely-deconditioned patients with COPD (Vivodtzev et al., 2008). Endurance (combined or not with resistance) training, however, did not confer the same improvement in voluntary activation in milder patients with COPD (Mador et al., 2001, 2004). It has also been suggested, from animal studies, that exercise training may induce neuroplasticity in brains areas responsible for the regulation of sympathetic activity (Mueller, 2007a). For instance, it has been shown that the rostral ventrolateral medulla was less sensitive to sympatho-excitation in exercise-trained rats compared to their sedentary counterparts (Mueller, 2007b). This might have implication for patients in whom impairments in cerebral hemodynamics may arise, at least in part, from increased sympathetic activity (e.g., those with CHF; Brassard and Gustafsson, 2016). Combining exercise training with acute (e.g., O_2_ supplementation) or chronic (e.g., CPAP) interventions might also prove relevant to lessen central abnormalities in respiratory disorders. Hyperoxia might be specifically relevant to patients with severe exertional hypoxemia (e.g., those with advanced COPD or *f*-ILD) to reverse any component of central fatigue, potentiating the beneficial effects of exercise training (Marillier et al., 2021a). The use of CPAP over sufficient periods of time (Canessa et al., 2011; Castronovo et al., 2014), alone or in association with exercise training, might have synergistic effects on central abnormalities induced by OSA. Future studies may also examine whether ameliorating neuromuscular abnormalities are paralleled by (or, perhaps, due to) brain structural changes (using imaging techniques such as magnetic resonance imaging; Macey et al., 2008) or changes in brain “health” markers, e.g., higher brain-derived neurotrophic factor (Hsu et al., 2021) and/or lower calcium-binding protein B (Alexandre et al., 2016). This may include animal studies mimicking cardiorespiratory conditions and investigating, for instance, micro- or macro-structural neuromuscular abnormalities/improvements and related functional capacity (Kapchinsky et al., 2018; Wafi et al., 2019). Importantly, any study aimed at improving central fatigue in patient populations should assess whether such changes are translated into a lower burden of symptoms and greater ability to carry on the activities of daily life.

## Conclusion

To date, only a few studies have objectively established the contribution of central and/or supraspinal mechanisms in limiting the tolerance to physical exertion in major cardiorespiratory diseases (Figure 6). This state of affairs largely stems from the challenges in establishing an appropriated experimental setting to get insights into the presence and etiology of neuromuscular fatigue in frail patients. A multidisciplinary approach comprising the assessment of brain structure, neuromuscular function, functional capabilities, and symptoms may prove valuable to uncover the contributory role of central mechanisms in their exercise limitation. Whether any amelioration in neuromuscular abnormalities (e.g., with rehabilitative exercise training) do translate into improved functional capacity and better health-related quality of life remains unclear at this point in time. Owing to the fact that improvements in exercise tolerance after exercise reconditioning are typically lost over time in patients with chronic cardiorespiratory diseases (Holland et al., 2008; Rochester and Spruit, 2017; Berger et al., 2021), future research should also focus on the best strategies to promote sustainable behavioral changes toward a more active lifestyle.

## Author Contributions

MM and MG conceived the article. MM reviewed the relevant literature on the topic and drafted the manuscript. MM, MG, A-CB, SV, and JN were involved in the interpretation of the presented data and provided critical feedback to shape the final version of the manuscript. All authors have read and approved the final version of the manuscript and agree to be accountable for all aspects of the work in ensuring that questions related to the accuracy of integrity of any part of the work are appropriately investigated and resolved. All persons designated as authors qualify for authorship, and all those who qualify for authorship are listed. All authors contributed to the article and approved the submitted version.

## Conflict of Interest

The authors declare that the research was conducted in the absence of any commercial or financial relationships that could be construed as a potential conflict of interest.

## Publisher’s Note

All claims expressed in this article are solely those of the authors and do not necessarily represent those of their affiliated organizations, or those of the publisher, the editors and the reviewers. Any product that may be evaluated in this article, or claim that may be made by its manufacturer, is not guaranteed or endorsed by the publisher.
